# Monochromic light reduces emergence delirium in children undergoing adenotonsillectomy; a double-blind randomized observational study

**DOI:** 10.1186/s12871-021-01435-1

**Published:** 2021-09-08

**Authors:** Adam C. Adler, Brian H. Nathanson, Arvind Chandrakantan

**Affiliations:** 1 Department of Anesthesiology, Perioperative and Pain Medicine, Texas Children’s Hospital, 6621 Fannin Street, Houston, TX USA; 2 Baylor College of Medicine, Houston, TX USA; 3 OptiStatim, LLC, Longmeadow, MA USA

**Keywords:** Blue light, Monochromatic light, Delirium, Pediatric, Emergence

## Abstract

**Background:**

Emergence delirium (ED) is common in pediatric anesthesia. This dissociative state in which the patient is confused from their surroundings and flailing can be self-injurious and traumatic for parents. Treatment is by administration of sedatives which can prolong recovery. The aim of this study was to determine if exposure to monochromatic blue light (MBL) in the immediate phase of recovery could reduce the overall incidence of emergence delirium in children following general inhalational anesthesia.

**Methods:**

This double blinded randomized controlled study included patients ages 2–6 undergoing adenotonsillectomy. Postoperatively, 104 patients were randomization (52 in each group) for exposure to sham blue or MBL during the first phase (initial 30 min) of recovery. The primary outcome was the incidence of emergence delirium during the first phase. We also examined Pediatric Anesthesia Emergence Delirium (PAED) scores throughout the first phase.

**Results:**

Emergence Delirium was reported in 5.9% of MBL patients versus 33.3% in the sham group, p = 0.001. Using logistic regression adjusting for age, weight, gender, ASA classification and PAED scores provided an adjusted relative risk ratio of 0.18; 95% CI (0.06, 0.54); p = 0.001 for patients in the MBL group. 23.5% of MBL patients versus 52.9% of sham patients had either ED or PAED scores of 12 or more throughout the first phase of recovery, p = 0.002. This produced an adjusted relative risk of 0.46, 95% CI (0.29, 0.75), p = 0.001.

**Conclusions:**

Monochromatic blue light represents a non-pharmacologic method to reduce the incidence of emergence delirium and PAED scores in children.

**Trial Registration:**

#NCT03285243 registered on 15/09/2017

**Supplementary Information:**

The online version contains supplementary material available at 10.1186/s12871-021-01435-1.

## Introduction

Emergence agitation (EA) and emergence delirium (ED) are frequently encountered in children emerging from general anesthesia. The incidence of EA/ED ranges between 10–80% depending on the scale used for assessment. Emergence delirium occurs most commonly in younger children (3–7 years) and following ophthalmology and otorhinolaryngology procedures [1]. Emergence delirium specifically is a dissociative state in which the patient is confused and dissociated from their surroundings and usually occurs within the first 30 min of recovery from anesthesia [2]. Sedatives (especially benzodiazepines and dexmedetomidine) have been shown to reduce the incidence of emergence delirium; however, their use often results in delayed discharge from the post anesthesia care unit (PACU) [2, 3]. This phenomenon often coincides with restlessness and flailing. These actions can be self- injurious and result in disruption to tubes, lines, drains, and is quite upsetting for parents and staff. Treatment of ED involved pharmacologic agents to induce sedation, most commonly propofol, benzodiazepines, or dexmedetomidine.

The primary aim of this study was to determine if exposure to monochromatic blue light in the immediate phase of recovery (defined as the first 30 min) could reduce the overall incidence of emergence delirium in children following general inhalational anesthesia. The secondary aim was to assess the effect of MBL on nursing assessment of ED using the Pediatric Emergence Delirium Scale (PAED) at varying points during the initial recovery phase [1]. Our hypothesis was that the patients receiving MBL would have lower incidence of ED and be less likely to experience either ED or have a PAED Scale score of 12 or more throughout the study period (initial 30 min in PACU).

## Methods

This study was conducted at Texas Children’s Hospital between November 3, 2017 and June 2, 2020. The study was approved by the ethical committee at Baylor College of Medicine on 12/09/2017 (IRB#: H#39,878; Principal investigator Adam C. Adler, MD) with written informed consent obtained from parents or legal guardians participating. The trial was registered at www.clinicaltrials.gov #NCT03285243, Principal investigator Adam C. Adler, MD, on 15/09/2017 prior to patient enrollment. All methods were carried out in accordance with relevant guidelines and regulations and with CONSORT recommendations.

Children 2–6 years old with American Society of Anesthesiologists (ASA) physical classification category of 1,2 (3 when due to sleep apnea related to the procedure with no other co-morbidities) were eligible for inclusion if they were undergoing adenotonsillectomy under general anesthesia.

Children receiving premedication with midazolam or dexmedetomidine were excluded owing to the reduction in emergence delirium with these agents. Other exclusion criteria were patients with known ocular disorders, migraines, seizures, psychiatric or behavioral health conditions, pre-operative anxiety, developmental delay, and patients taking stimulants for appetite or attention deficit hyperactivity disorders. Patients having received midazolam, dexmedetomidine, or ketamine either preoperative or intraoperatively were also excluded. Patients unable to be transferred to PACU immediately following extubation were also excluded.

Patients are brought to the operating room either walking, in a toy car and occasionally with child life accompanying. At our institution, parental presence is rarely performed and only on insistence from the family.

### Anesthetic conduct

All patients underwent routine anesthetics with inhalation induction (sevoflurane 8% with nitrous oxide in oxygen 70%/30%) and maintenance was with sevoflurane. All patients were intubated and received opioids at the discretion of the anesthesiologist. Propofol was given prior to intubation if deemed necessary by the anesthesiologist. Midazolam, ketamine and dexmedetomidine (pre or intraoperatively) were not administered. Intravenous ondansetron and dexamethasone were administered to all patients intraoperatively. At the conclusion of the procedure, the head of the bed was turned 90 degrees to the standard position and the patient extubated. Patients were immediately transferred to a stretcher and brought directly to PACU with blow by oxygen using a modified Jackson Rees (King Systems, Noblesville, IN) and facemask. Any patient unable to be immediately transferred to PACU following extubation (e.g. PACU recovery hold delay) was excluded. In line with institutional practice for adenotonsillectomy, all patients were extubated under deep general anesthesia and immediately brought to the post-anesthesia recovery unit (PACU). This was done to ensure that all emergence started uniformly in all patients as well as the assessment of the baseline PAED score from the time sevoflurane was discontinued.

### Randomization

Randomization was performed using individually sealed envelopes assigning patients to group A or group B designating the study group (MBL) and the control group (Sham), respectively. Envelopes were sorted to allow for random allocation. The envelope was opened immediately on arrival to the PACU and the lightbox was set to A or B accordingly. To avoid influencing intraoperative practice, randomization was performed immediately on entry to the PACU. To simulate normal practice conditions, the PACU nurses were instructed to report subjective occurrence of ED to the anesthesia attending and treat per their standard practice. In addition, the PACU nurses were also asked to complete the Pediatric Anesthesia Emergence Delirium (PAED) scale with the baseline performed on arrival to the PACU (Supplemental Table 1) [4]. We chose to have the PACU nurse perform the PAED scale vs. a dedicated research team member to simulate normal practice conditions. To reduce bias, all PACU nurses were kept blinded to the patient grouping.

### Study conduct and patient assessment

The lightbox Draeger Phototherapy 4000 (Draeger Medical, Lübeck, Germany) was placed behind the head to the bed directly over the patient at 90 degrees and 12 to 18 inches above the face. All patients were positioned supine for maximal exposure. Patients were immediately randomized to study group or placebo and the light was started within 1 min of entry to the PACU. To allow for concealed allocation, control patients were exposed to sham blue light that was not monochromatic but contained all wavelengths within the visible spectrum (3000 K) (i.e. it appeared blue due to an outer coating on the bulb) (Osram Dulux, Augsberg, Germany). To confirm that this coating simply tinted the light blue without altering the light mechanics, a GaP photodiode radiometer (Solarmeter, Glendale PA) was used prior to study initiation. The study group was exposed to monochromatic light with wavelength peak 460 nm (range 400–500) and 7100 K (Draeger Medical, Lübeck, Germany). To ensure irradiance was maintained throughout the study, irradiance of the bulbs was checked yearly. To maintain blinding, both the sham and experimental bulbs were within the same lightbox.

All PAED scores were taken on entry to the PACU (baseline) and at 10, 20, and 30 min for each patient by dedicated pediatric PACU nursing staff. PAED scores were not recorded if the patient was awake and appropriate or experienced emergence delirium during this time as the scores no longer had clinical relevance since the study outcome is now definitively known. The exposure to the light was also for 30 min or until the patient was awake and appropriate or had emergence delirium. In addition to recording the PAED scores, the nurses were also asked to report if the patient experienced ED. If the PACU nurse observed the patient to have ED, they notified the anesthesiologist or covering anesthesiologist to provide pharmacologic treatment at their discretion.

### Statistical analysis

To describe the cohort, we calculated means and standard deviations (SD) or counts and frequencies as appropriate for the variables collected. We compared the MBL and Sham groups using the Student’s t-test for continuous variables (e.g., age, PAED score) and the chi-square test for categorical variables (e.g. % female, ASA category), except when a cell count was < 5, in which case we used the Fisher’s exact test. For baseline comparisons, we calculated absolute standardized differences instead of p-values. Missing data for the PAED scores over time indicated a patient woke up or experienced emergence delirium and so no imputation was performed. There was no other missing data in the variables. To adjust for any remaining confounding among the variables collected, we calculated a logistic regression model with the outcome being emergence delirium and a second logistic regression model with the outcome being either ED or the patient had a PAED score of 12 or more at time periods 10, 20, and 30 min. Both regression models used as independent predictors the following variables: treatment group, age, weight, gender, and ASA category. Robust standard errors were derived to account for any within-cluster correlation at the individual nurse level given the possible serial correlation of outcomes within each nurse caring for his or her patients [5].

An a priori calculation of sample size was done using an incidence of 40% of emergence delirium in similar patient population [6]. We assumed an absolute reduction of 25% (ie, 40% to 15%) in incidence of emergence delirium to be clinically significant. To detect this difference at 80% power with a two-sided alpha of 0.05, required a sample size of 98 participants. All analyses were done using Stata/MP 15.1 for Windows (StataCorp LLC, College Station, TX). All p-values < 0.05 were considered statistically significant.

## Results

**Fig. 1:**
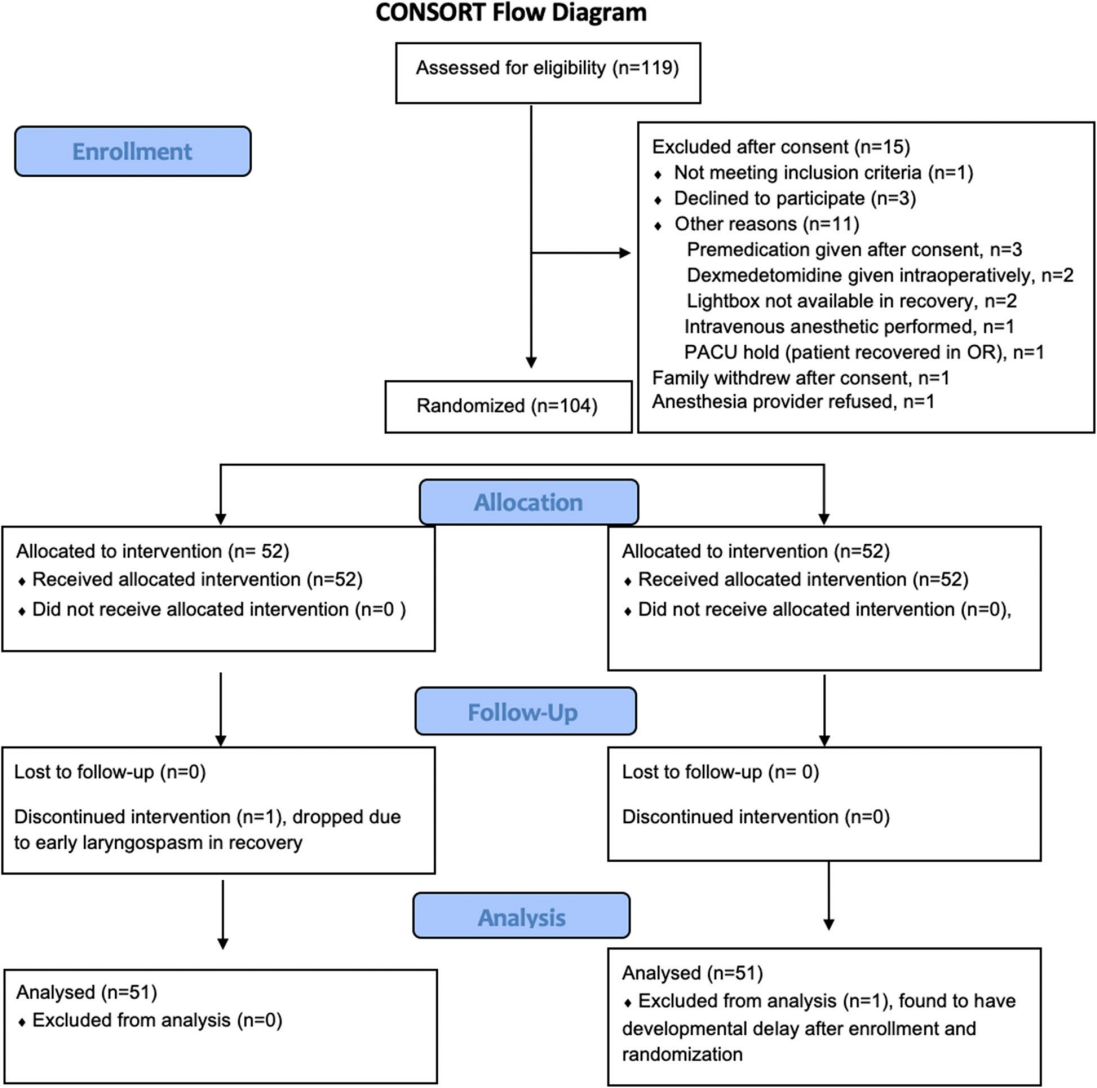
Flowchart of patient enrollment. PACU = post anesthesia care unit

A total of 119 patients were enrolled with 104 patients undergoing randomization and 102 completing the study and included in the final analysis (Fig. 1). The patient characteristics between the MBL (treatment) and Sham (control) groups are summarized in Table 1. There was no difference identified in demographic data between groups. For the primary outcome, emergence delirium was reported in 5.9% of MBL patients versus 33.3% of the Sham patients; *p* = 0.001 (Table 2). Logistic regression adjusting for age, weight, gender and ASA classification and nurse recording the PAED provided an adjusted relative risk ratio of 0.18; 95% CI (0.06, 0.54); *p* = 0.001 for patients in the MBL group (Table 3).

**Table 1:** Patient demographics for children undergoing adenotonsillectomy by study group. ASA = American Society of Anesthesiologists

Variables	Control Group (sham); *N* = 51	Study Group (Monochromatic Blue light); *N* = 51	Absolute Standardized Differences
Age (Mean, SD)	4.2 (1.4)	4.7 (1.3)	0.40
% Female (n, %)	24 (47.1%)	24 (47.1%)	0.00
Weight (kg) (Mean, SD)	20.3 (7.2)	21.8 (7.0)	0.21
ASA physical class (n, %)			
1	0 (0%)	4 (7.8%)	
2	40 (78.4%)	36 (70.6%)	0.42
3	11 (21.6%)	11 (21.6%)	
Baseline (time = 0) Pediatric Anesthesia Emergence Delirium Scale Score (Mean, SD)	12.0 (0)	11.9 (0.7)	0.20

**Table 2:** Patient assessment of emergence delirium. PAED = Pediatric Anesthesia Emergence Delirium

Variables	Sham Group (control); *N* = 51	Monochromatic Blue Light Group (treatment) *N* = 51	*P*-value
**Outcomes**			
Patient had Emergence Delirium (n, %)	17 (33.3%)	3 (5.9%)	0.001
Patient had Emergence Delirium or a PAED Scale Score of 12 or more for 30 min after Baseline (n, %)	27 (52.9%)	12 (23.5%)	0.002
PAED Scale Score over time (after Baseline)			
Time = 10 min (Mean, SD)	*n* = 50; 12.8 (2.8)	*n* = 47; 12.5 (2.0)	0.592
Time = 20 min (Mean, SD)	*n* = 40; 12.9 (4.4)	*n* = 38; 11.6 (4.5)	0.185
Time = 30 min (Mean, SD)	*n* = 25; 10.9 (5.2)	*n* = 27; 6.6 (5.9)	0.007
PAED score recorded at all 3 periods (n, %)	25 (49.0%)	27 (52.9%)	0.692
Percent change from baseline PAED score to last recorded PAED score (Mean, SD)	-1.7% (46.5)	-26.8% (49.6)	0.012

**Table 3:** Multivariable Logistic Regression Model Identifying Factors Involved In Experiencing Emergence Delirium. ASA = American Society of Anesthesiologists

Variable	Relative Risk Rat	95% Confidence Interval	*P*-value
Study Group (Baseline Category = Control Group)	0.18	(0.06, 0.54)	0.001
Age (per year)	0.88	(061, 1.26)	0.470
Weight (per kg)	1.02	(0.96, 1.09)	0.554
Female Sex (Baseline Category = Male)	1.45	(0.83, 2.56)	0.186
ASA Category			
1 or 3	1		-
2	0.70	(0.29, 1.68)	0.447

**Fig. 2:**
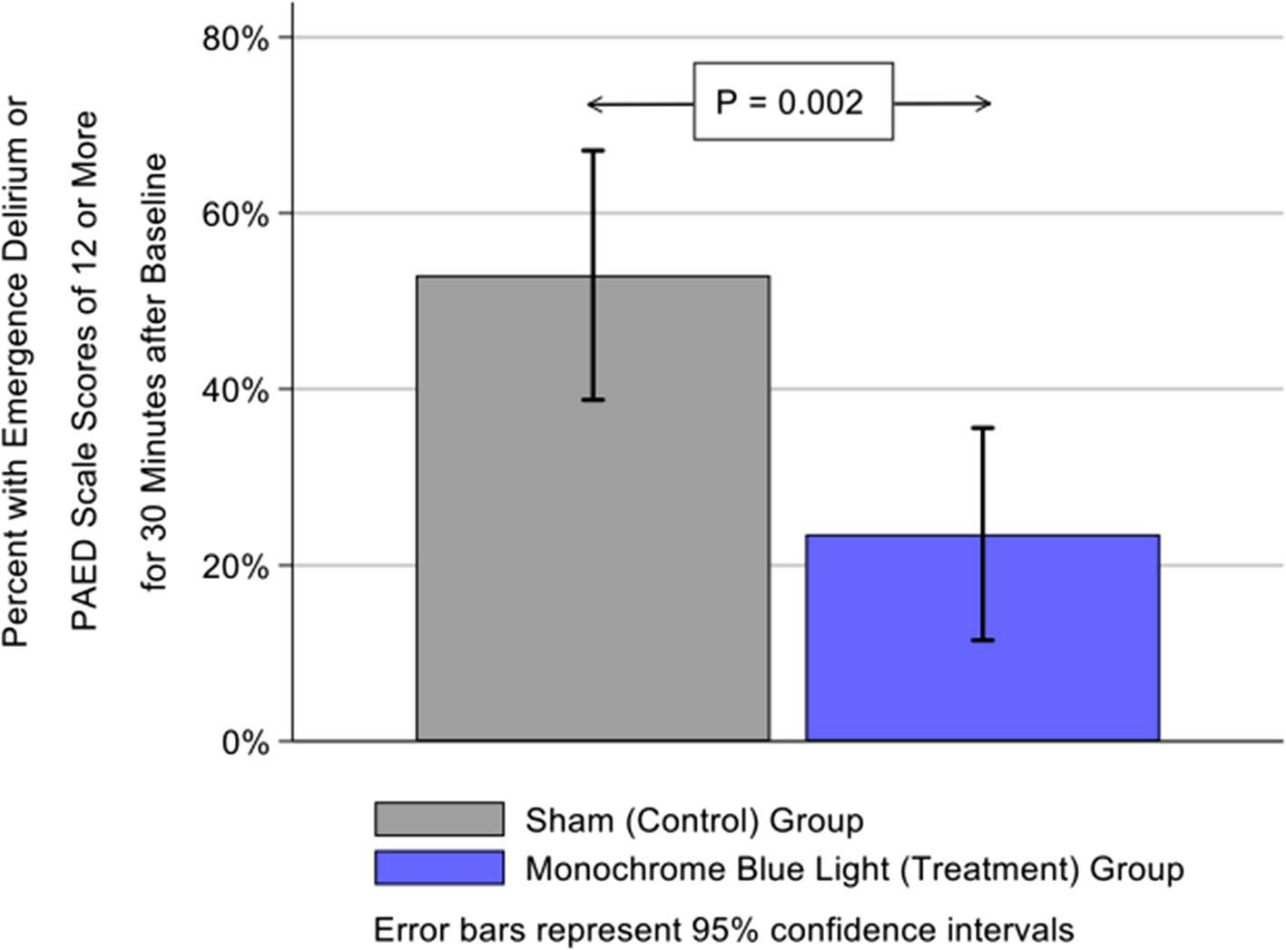
Percent change in Pediatric Anesthesia Emergence Delirium (PAED) score between patients exposed to monochromatic blue light (study) and those exposed to polychromatic sham blue light (control)

When examining if a patient experienced ED or had a PAED score of 12 or higher at the time periods of 10, 20, and 30 min, 23.5% of MBL had this outcome versus 52.9% of the Sham group, *p* = 0.002 (Fig. 2). This produced an adjusted relative risk of 0.46, 95% CI (0.29, 0.75), *p* = 0.001 (Supplemental Table 2).

The mean PAED scores at time intervals of 10 and 20 min were similar between groups. For example, the mean (SD) observations at 20 min was 11.6 (4.5) versus 12.9 (4.4) for the MBL and Sham groups, respectively. However, at time = 30 min from arrival to PACU, the MBL group had a lower mean PAED score compared with controls (6.6 (5.9) vs. 10.9 (5.2); *p* = 0.007) respectively and we note that these values exclude patients who had ED or awoke before this time. Patients exposed to MBL had a significant reduction in PAED scores from baseline to their last recorded PAED score when compared with the Sham group (-26.8% vs. -1.7%; *p* = 0.012) respectively (Table 2).

## Discussion

Our data shows that exposure to monochromatic blue light in the immediate recovery phase following general inhalational anesthesia reduces the incidence of emergence delirium. The incidence of ED in our control group was similar to the reported incidence in children undergoing adenotonsillectomy [7, 8]. However, for patients in the MBL group, the incidence was significantly less than what was observed in the Sham group (5.9% versus 33.3%). We also observed if a patient experienced ED or had a PAED score of 12 or higher at the time periods of 10, 20, and 30 min, 23.5% of MBL had this outcome versus 52.9% of the Sham group, p = 0.002. We also noted that the differences in the PAED scores were most pronounced after the 20-min mark of the study. We theorize that this is due the time of emergence in patients for which deep extubation is performed.

Monochromatic light has been shown to stimulate wakefulness and alertness, reduce reaction times and enhance cognition [9–13]. Functional MRI studies during MBL exposures correlated with increased hippocampus, thalamic and amygdala activity as well as increased activity within the frontal gyrus, and a bilateral area of the brainstem consistent with activation of locus coeruleus [11, 13]. Electroencephalogram (EEG) analysis during daytime exposure to MBL of 460 nm revealed reduced power density of theta and low frequency alpha waves while nighttime exposure was associated with reduced delta wave power and increased high frequency alpha power [10].

There is limited patient data on EEG findings in children experiencing ED [14]. Martin et al. described EEG recordings in children during emergence from sevoflurane-based anesthetics including five patients during ED [14]. During emergence there is delta frequency slowing and frontally dominant alpha activity (indeterminant phase) preceding a prolonged state of low-voltage fast frequency activity prior to appearance of either normal wake or sleep EEG patterns. In children with ED, a variable indeterminant phase was observed prior to return of normal wake or sleep patterns. They observed atypical EEG patterns following the indeterminant phase during the delirium. For reasons unknown, ED is associated with arousal from the indeterminant phase prior to the return of normal EEG patterns. Additionally, increased frontal connectivity was observed in patients with ED immediately after termination of the sevoflurane. Based on the effect of MBL on EEG as well as the change to activity during ED, we postulate that exposure to MBL may enhance the return to the normal wake or sleep patterns during early emergence from sevoflurane anesthesia.

While the robust, double blind study design and rigorous statistical methods are study strengths, the analysis does have limitations. The assessment of ED as well as the PAED scale is a subjective clinical determination and may also suffer from the Hawthorne effect. However, to reduce the Hawthorne effect, we used a sham blue light with all PACU nurses completing the assessments remained blinded to the monochromatic and sham group designation. We also adjusted for the nurse completing the ED assessment (*n* = 36). We did not control for intraoperative intravenous acetaminophen administration. However, at our institution, patients undergoing outpatient adenotonsillectomy often receive oral acetaminophen with oxycodone and only two patients received intraoperative acetaminophen (15 mg/kg) both in the sham group (1 patient had emergence delirium and one did not). Another limitation is that we did not control for intraoperative opioid use as narcotics which may result in sedation and effect the onset of ED [15]. While most patients received opioids at the time of intubation, the mean (SD)[IQR] time from last opioid to extubation was 32.2 min (13.1) [25–40] and 24.5 min (12.8) [15–30] in the MBL and sham groups respectively. This study did not record EEG activity during emergence from anesthesia; rather serving as a concept to identify if a reduction of ED was present. Finally, while not a limitation of the analysis per se, we acknowledge that our study design does not elucidate the hypothesized mechanism of action for the observed reduction in ED. Additional research to examine this hypothesized mechanism would be of interest.

In conclusion, this double blind-randomized observation study found that MBL when compared with significantly reduced the incidence of emergence delirium in children following sevoflurane anesthesia. Compared with sham, MBL was associated with reduced emergence delirium as lower mean PAED scores. Exposure to MBL during the early emergence phase from sevoflurane anesthetics may be a potentially non-pharmacologic intervention that reduces ED in children.

## Supplementary Information

**Additional file 1.**

## Data Availability

The datasets generated and/or analyzed during the current study are not publicly available due privacy but are available from the corresponding author on reasonable request.

## Abbreviations

MBL: Monochromatic blue light (MBL)
PAED: Pediatric Anesthesia Emergence Deliriums
ED: Emergence delirium
EA: Emergence agitation
PACU: Post anesthesia care unit
ASA: American Society of Anesthesiologists
SD: Standard deviations
EEG: Electroencephalogram
