# Supplementary material for: Monochromic light reduces emergence delirium in children undergoing adenotonsillectomy; a double-blind randomized observational study
Source: BMC Anesthesiol. 2021 Sep 8;21:217. doi: 10.1186/s12871-021-01435-1 (PMC8424999; doi:10.1186/s12871-021-01435-1)
Supplement: Supplementary file 1 — Additional file 1. [file 12871_2021_1435_MOESM1_ESM.docx]

Supplemental Table 2: Multivariable Logistic Regression Model for the outcome; Emergence Delirium or PAED Score of 12 or More throughout the First Phase (30 Minutes). ASA=American Society of Anesthesiologists

| Variable | Relative Risk | 95% Confidence Interval | P-value |
| --- | --- | --- | --- |
| Study Group (Baseline Category = Control Group) | 0.46 | (0.29, 0.75) | 0.001 |
| Age (per year) | 0.86 | (0.67, 1.11) | 0.236 |
| Weight (per kg) | 1.02 | (0.98, 1.07) | 0.353 |
| Female Sex (Baseline Category = Male) | 1.72 | (1.20, 2.47) | 0.005 |
| ASA Category |  |  |  |
| 1 or 3 | 1 | - | - |
| 2 | 0.94 | (0.43, 2.05) | 0.910 |
